# Management of chronic non-cancer pain by primary care physicians: A qualitative study

**DOI:** 10.1371/journal.pone.0307701

**Published:** 2024-07-26

**Authors:** Léa Rufener, Christina Akre, Pierre-Yves Rodondi, Julie Dubois

**Affiliations:** 1 Faculty of Science and Medicine, University of Fribourg, Fribourg, Switzerland; 2 Department of Epidemiology and Health Systems, Center for Primary Care and Public Health (Unisanté), University of Lausanne, Lausanne, Switzerland; 3 Faculty of Science and Medicine, Institute of Family Medicine, University of Fribourg, Fribourg, Switzerland; Manchester Metropolitan University - Aytoun Campus: Manchester Metropolitan University, UNITED KINGDOM OF GREAT BRITAIN AND NORTHERN IRELAND

## Abstract

Chronic non-cancer pain is a highly prevalent health issue with personal and societal consequences. Patients suffering from chronic non-cancer pain are mainly cared for by primary care physicians, but research shows that the latter perceive treating chronic pain as difficult. This qualitative descriptive study aimed to explore how primary care physicians in Switzerland manage patients with chronic non-cancer pain and what factors influence patient management. Data were collected through semi-structured interviews amongst primary care physicians in the German speaking part of Switzerland. A thematic analysis of the interviews allowed to identify four main themes: Investigation of chronic pain; patient-provider relationship; patient characteristics, and medical recommendations. These themes were closely interconnected and influenced each other. Physicians not only enquired about the origin of pain but also about the patients’ beliefs and expectations towards it. They stressed the role of communication in fostering a good patient-physician relationship and to help patients cope with their pain. In addition to purely medical considerations, the psychological, social and economic situation of their patients and their possible impacts on the management of chronic non-cancer pain played a crucial role when recommending a treatment. This study highlighted the complexity of chronic pain management, which entails that primary care physicians need to figure out a unique strategy for each patient. By integrating patients’ values and beliefs, as well as socioeconomic aspects, primary care physicians are in a position to take the lead in chronic non-cancer pain management. However, considering the burden of this disease, more continuous medical education on chronic pain is needed for primary care physicians, especially to better take into account the social determinants of pain.

## Introduction

Chronic non-cancer pain (CNCP) is frequent and burdensome, with a 1-month prevalence of 19% in the adult population in Europe [1]. In the International Classification of Diseases-11, chronic pain is defined as “pain that persists or recurs for longer than three months” [2]. Within CNCP, low back pain (LBP) and neck pain are the leading causes of years lived with disability since 1990 [3]. Patients suffering from CNCP report that it impacts their physical and mental health, and thus, affects their ability to work, to perform daily activities, and to maintain social activities and personal relationships [1, 4]. Furthermore, the rate of unemployment and social assistance is higher in people with CNCP than in the general population [5, 6]. In addition, the direct and indirect socioeconomic cost of CNCP was found to be at least as high as the cost of conventionally prioritized health conditions such as cardiovascular disease and cancer [7, 8].

Primary care physicians (PCPs) are at the forefront of the treatment of CNCP: 70% of patients suffering from CNCP are being treated by PCPs [9]. PCPs thus play an important role in the long-term management of patients with chronic pain [10]. There is a wide range of treatments for CNCP including pharmacological, interventional, physical and psychological approaches, as well as complementary medicine approaches. In addition, in an attempt to treat CNCP efficiently, the prescription of opioids has increased over the last two decades and with it the rates of adverse effects, abuse, and addiction [11, 12]. For example, between 1985 and 2015, Switzerland has seen an 23-fold increase in opioid consumption, placing the country above European average in terms of total consumption per inhabitant [13]. Whatever the treatment chosen, they all provide only modest improvement in pain and pain-related functioning [8, 14]. In addition, due to the complex etiology of CNCP, a combination of different treatments is usually needed [8, 14]. Furthermore, PCPs perceived effectiveness of a treatment is sometimes contradictory with their prescribing behaviors. [15].

To understand how PCPs manage patients with CNCP, qualitative studies have explored attitudes and prescription behaviors of PCPs [16–18], particularly in the context of LBP [19–21] or opioids prescribing [22–24]. These studies have found multiple factors at different levels influencing pain management and treatment choice, such as the quality of the patient-provider relationship, patients’ and physicians’ beliefs, diagnostic uncertainty, patients’ socio-economic background, organizational constraints, and health policies. However, there are very few recent studies that have investigated how PCPs manage CNCP in general from investigation of pain to treatment choice, without focusing on a given treatment option or pain site. In addition, there are no data on how Swiss physicians manage CNCP and on whether they face similar constraints as those identified by the literature. Thus, the leading objective of this study was to explore how primary care physicians in Switzerland manage patients with CNCP and what factors influence patient management.

## Methods

### Study design

This is an exploratory qualitative descriptive study [25] using semi-structured interviews amongst PCPs in the German speaking part of Switzerland. As stated by Sandelowski [25], descriptive designs are “especially amenable to obtaining straight and largely unadorned (i.e., minimally theorized or otherwise transformed or spun) answers to questions of special relevance to practitioners and policy makers”.

### Study population

The study population were PCPs, with a practice in the German speaking part of the canton of Fribourg and the Basel region in Switzerland. To be included, PCPs had to meet the following inclusion criteria: 1) being currently working in a private practice in the cantons of Fribourg or Basel; 2) having treated at least one patient with CNCP over the past year; 3) being fluent in German. For this study, we defined CNCP as a pain lasting or recurring for more than three months, in accordance with the International Association for the Study of Pain [26].

### Data collection

An interview guide was developed based on topics previously identified in the literature about the management of CNCP and on discussions within the team (see S1 Appendix). A pilot interview was conducted with one PCP to ensure comprehensibility and coherence of the interview guide.

The recruitment was conducted using convenience and snowball sampling techniques. PCPs were looked up on the official index of the Swiss Medical Association (FMH) [27] and chosen according to the area where they practiced. PCPs were contacted by phone. PCPs interested in participating in the study were then sent an email containing further details about the conduct of the study, as well as a complete information letter attached. PCPs then called LR back in order to set a time and place for the interview.

The interviews were conducted in German by LR (first author, 4^th^ year medical student) after having been trained in their conduct by JD (last author, senior qualitative researcher in primary care). The face-to-face interviews took place between October 2019 and March 2020 at the practice or clinic where the participants worked. The interviewer introduced herself prior to the interviews and stated the study objectives. All participants were informed that this study was conducted as part of a master’s thesis in medicine. At the beginning of the interview each physician signed an informed consent form. During the interviews, PCPs were encouraged to tell case examples but asked to avoid patients’ identifiers. All interviews were audio recorded. The recordings were then transcribed verbatim by the interviewer (LR) in their original language. Only LR had access to the coding key linking the participant’s name with their interview recording and transcript in order to guarantee confidentiality. Any information that could identify a participant or his/her patients was removed from the transcripts. The transcripts were not returned to the participants.

The Cantonal Commission for the Ethics of Human Research (CER-VD) waived the need for formal ethics approval for this study as no health-related data were collected (Reference Req-2019-01258). However, all procedures performed in this study were in accordance with the Swiss Federal Act on Research involving Human Beings [28] and with the 1964 Helsinki Declaration [29] and its later amendments or comparable ethical standards.

### Data analysis

The interviews were thematically analyzed [30] with the use of a qualitative data analysis software (QDA miner lite v. 2.0.7). As a first step, the first two transcripts were coded separately by two researchers (LR and JD). After comparing their coding, the two researchers cooperatively developed a codebook which was then used by LR to label the content of all the following transcripts. The codebook was adapted iteratively as new codes emerged from the data. Several cross-checks of the coded transcripts were performed by JD to ensure validity and rigor of the coding. New codes, changes in the definitions of codes, and discrepancies in coding were discussed between LR and JD until reaching an agreement. Afterwards, all codes were compared, similar codes were merged, and codes were classified into larger themes and subthemes. Findings were then discussed within the research team, which comprised another senior qualitative researcher in public health (CA), and a physician and professor in primary care (PYR), allowing for multiple perspectives on the data. The analysis was both deductive and inductive: although the major themes were derived from the interview guide, attention was also paid to new aspects revealed by the interviews that were not present in the guide.

Our data are based on quotation from participants. LR, who is a German speaker and fluent in English, translated the quotations from German to English, with the help of JD. Finally, an additional reading was carried out by a CA, a native English speaker, to make sure the idiomatic meaning of statements of the participants was preserved. We used the Consolidated criteria for Reporting Qualitative research (COREQ) checklist to report our study [31].

## Results

A total of twenty primary care physicians were contacted of which ten (50%) agreed to participate in the study. The other ten refused mostly due to time constraints or because they had reached the end of their career. The mean age of the participants was 45.4 (SD 6.3). They were mostly female (N = 6; 60%) and had been in practice for a mean of 9.3 years (Range 1–23; SD 7.0).

The duration of the interviews varied between 18 and 42 minutes (mean: 29.2). Four main themes were identified during the analysis, namely: investigation of CNCP, patient-provider relationship, patient characteristics, and medical recommendations. An overview of the thematic structure of the results is provided in Table 1.

**Table 1:** Overview of the thematic structure of results.

Themes	Sub-themes
Investigation of CNCP	History taking
	Looking for specific causes
	Exploration of psychosocial dimensions
Patient-provider relationship	Fostering the patient-provider relationship
	Communicating with patients
Patient characteristics	Socioeconomic situation
	Patients’ attitude towards pain
Medical recommendations	Pharmacological treatments
	Non-pharmacological treatments
	Reassessment
	Coping strategies

CNCP: chronic non cancer pain

### Investigation of CNCP

#### History taking

Knowing as much as possible about the pain was fundamental to all interviewed PCPs as a first step for adequate pain management. All PCPs mentioned that history taking, including patient’s previous medical journey, comorbidities and comedication, was an important part of the investigation.

“*Then it is also interesting to know how they are limited by this pain. (…) And what investigations have they already made, what resources do they have to cope with this pain. And very important: what have they already tried themselves, have they already taken alternative medicine, have they already tried medications and therapies, and what has actually helped them and what has not.*”—PCP 3

#### Looking for specific causes

Almost all PCPs also wanted to find the origin of pain, if any, before treating it. In order to find or exclude specific causes of pain, most PCPs chose to do additional diagnostic tests, such as laboratory tests and imaging procedures, before moving on with a chronic management plan.

However, some PCPs underlined that with CNCP conditions, there was rarely a single and specific cause.

“*The problem with many patients with pain is that they don’t have a clear underlying cause (…), that it’s a diffuse pain symptom where you don’t know exactly where it’s coming from, it starts at one point and intensifies over time. All kinds of medication and tests are performed, and nothing is found.*”—PCP 8

Nevertheless, in the absence of a clear underlying cause, PCPs were committed to keep searching for a cause, out of fear of missing something important and to reassure the patient.

“*My feeling is that even if it sometimes seems pointless in the follow-up visits, you still look again to check if something has changed, it gives the patients the feeling that you care about them and it gives you the security that you don’t miss anything. That always remains in the background with chronic pain, that you don’t want to miss anything.*”—PCP 8

That same PCP, along with others, underlined that patients with no clear underlying cause for their pain often come back to their PCPs with requests for additional investigation to obtain a diagnosis. Even if these additional investigations are contrary to clinical recommendations (e.g, recommendations discouraging the multiplication of diagnostic tests in the absence of red flags), PCPs consider the importance of maintaining a good relationship with their patients when deciding whether to undertake these investigations.

“*Most of the time, they [the patients] are the ones who come back again and again and need some kind of investigation. And then it’s a matter of weighing up whether to do something again to reassure the patients or not. And that is very difficult, according to the guidelines you should of course never do anything [more], but it is very difficult to distance yourself from the patients. Because otherwise they simply run to the next [physician], and then the big spiral starts all over again, (…) so that eventually you investigate a bit more…*”–PCP 8

#### Exploration of psychosocial dimensions

Most PCPs highlighted the importance of exploring the patient’s psychosocial situation and social environment in order to provide adequate management.

“*Also, with the whole psychosocial situation in the background, which is of course also interesting to find out. Why do they have this pain in particular, are there perhaps parallels in the family or among the relatives or somewhere else in the environment? What does this pain mean to them?*”—PCP 7

Investigating the patients’ interpretation of their pain also helped to situate the patients’ beliefs and expectations, as well as identify psychological factors that might influence pain, and was thus crucial for comprehensive pain care.

“*(…) What I want to say is that the patient’s interpretation of their pain is very important for pain processing, which is why it is important for me to know where they think it comes from (…). It is [also] important for me that I know something more about their history, where this person comes from. Because with chronic pain we also partly have to deal with psychological factors that have to be taken into account.”.*”—PCP 6

### Patient-provider relationship

#### Fostering the patient-provider relationship

Most interviewees stated that their relationship with the patient is crucial for the outcome of a therapy and considered that they have a pivotal role in the management of their patients’ pain, in particular as guarantors of the continuity of care.

“*That’s also the good thing, as a general practitioner you accompany people, you don’t just have one conversation, you see the people again. And with time, you can convey certain messages to them.*”—PCP 1

Most PCPs also highlighted the importance of a trusting patient-physician relationship where patients can confide in their PCPs and rely on their opinion.

“*That is actually perhaps the most important thing about the whole thing, that they (…) feel understood and that they know that they can and should come back at any time if they are not OK. And it is also very important that one should try not to say anything negative about any therapist or type of therapy to the patient, which they thought would be good. Because then there is a great danger that the relationship, the relationship of trust, will be destroyed.*”—PCP 3

#### Communicating with patients

All PCPs agreed that communication plays an important part in CNCP management. Properly communicating about the condition was crucial but also challenging to some PCPs.

“*With patients with chronic pain, conversations are the most important thing. I can’t add much value with an X-ray or ultrasound, but I can with a conversation.*”—PCP 2

In particular, PCPs felt that patients needed to understand the causes and mechanisms of chronic pain in order to be able to adequately cope with it. For example, in response to the question of why new patients with chronic pain were often unhappy with their previous treatments after visiting numerous physicians and pain specialists, one PCP answered:

*“Because it doesn’t work and because perhaps no one has ever spoken plainly and explained everything to them. Has the patient understood what the cause of the pain is and how the process of chronic pain comes about? Has it been explained to them? I think the more informed the patient is, the better they can deal with it and also accept it as–maybe–fate.”—PCP 6*

Some PCPs mentioned time management as a difficulty when communicating about CNCP. They considered that patients with CNCP need longer consultations, in order to receive proper explanations on how their pain is dealt with and be reassured. A few PCPs specifically mentioned difficulties in verbal and cultural communication, where time restraints also complicate the matter.

“*When you can hardly communicate with patients, it becomes difficult. It requires more patience. The communication barriers can be linguistic, but they can also be cultural, in the sense that the patient may not be able to listen to a woman at all. But in both cases I have the feeling that it can be overcome with enough patience. You might need five conversations where otherwise you only need one.*”—PCP 2

### Patient characteristics

Patient management was also influenced by characteristics, real or perceived, specific to the patients’ personal situation (professional, financial) or, less often, to their attitude towards pain. Some of these were seen as obstacles to effective CNCP management.

#### Socioeconomic situation

Socioeconomic factors were often cited. Some PCPs considered how CNCP impacts the patients’ ability to work. They underlined how, in some cases, it was difficult to adapt the work so that a person suffering from CNCP would be able to perform it. For example, a PCP explained that, depending on the patient’s occupation, advice to avoid heavy loads is not always possible to apply in reality.

“*People who work in the construction industry, or as a warehouse worker, or on a farm, or who have other physically demanding work. It is often extremely difficult to find a way of reorganizing their work routine in a way that is easy on the back but does not lead to unemployment.*”—PCP 2

Most PCPs also took the patient’s financial resources into account before recommending a treatment. Indeed, while most conventional therapies are covered by the patient’s basic mandatory health insurance, other treatments, notably complementary medicine treatments, are only covered by optional supplemental insurances or paid out-of-pocket by the patients. This sometimes led PCPs to first propose treatments that would be reimbursed rather than suggesting ones that patients would have to pay for.

*“But the most common prescription is for physiotherapy (…). Why? Not because physiotherapy is the best, but because (…) it is part of the basic [health] insurance. And so even if (…), for example, acupuncture would make more sense, we often do physiotherapy first.*”—PCP 3

#### Patients’ attitude towards pain

As shown in the theme “Patient-Provider relationship”, PCPs wanted their patients to understand the causes and mechanisms of their chronic pain to better cope with it. However, this led a minority of PCPs to suggest that suffering from CNCP may be related to the patient’s attitude towards pain. For example, to the question why patients’ understanding of the causes and mechanisms of chronic pain may play a role in making the therapy work better, one PCP answered:

“*Cognition influences (…) healing processes, that’s how it is. So, people who absolutely want to work again soon and be fit, usually they also get fit again and they don’t have chronic pain.*”—PCP 1

Perceptions about their patients’ attitudes towards pain led a few PCPs to suspect that some patients may have a secondary gain from their pain.

“*Well, and especially in the case of chronic pain problems, there is of course always the matter of disability insurance or the workplace situation, where you also have to keep an eye on the secondary gain from the illness (…). I think there comes a time when the pain should not go away anymore, because otherwise there could be disadvantages. In other words, a disability insurance pension that is no longer paid, (…) a job that would have to be taken up again (…).*”—PCP 7

### Medical recommendations

Several PCPs agreed that a lot of experience is needed to provide adequate care for patients with CNCP. The PCPs personal awareness of treatment options and their experience with them influenced what they prescribe. For successful referral, personal network with therapists and clinics was deemed important. Most agreed that if they were not sufficiently familiar with a specific treatment, they would refer patients to other professionals,

“*The ones [treatment modalities] I don’t prescribe, that’s what I don’t know, and I refer them to a pain center. These are mainly (…) these peripheral nerve blocks, these morphine pumps, I know too little about.*”—PCP 4

Similar to the patients’ request for additional investigations to obtain a diagnosis, some PCPs mentioned that patients with CNCP sometimes have specific demands for a treatment, whether pharmacological or non-pharmacological. After considering the risks and benefits of a requested treatment, and if it would not harm the patient, most PCPs were not opposed to prescribing it.

“*I then ask why they think that’s good, what their experience has been. And if that’s a medication where I feel it won’t harm the patient and may well be appropriate, then I don’t see a problem with trying that.*”—PCP 6

In turn, some PCPs mentioned that if a patient really didn’t want a certain treatment, they wouldn’t force that.

“*And if someone says from the start, ‘that’s not going to help me’, then that’s usually the case, because it’s already rejected from the start in a certain way. That’s why you always have to ask or discuss together what makes sense or what doesn’t.*”—PCP 9

#### Pharmacological treatments

All PCPs said that they prescribed pharmacological treatments for CNCP. For most, conventional non-opioid analgesics were the first treatment option that they provided.

PCPs also mentioned other substances such as antidepressants and anticonvulsants that are known to have a positive effect on some kinds of pain. Some PCPs acknowledged the effectiveness of antidepressants, as pain-modulating, or helping to take the focus off the pain. But most of them said that in the patients’ experience, taking antidepressant medication was still stigmatized, and therefore many patients refused it.

“*The problem is often when you utter ’antidepressants’, even if you explain to patients in detail that it’s not for depression, it’s for pain, it’s often the case that they then just don’t take it or don’t want to.*”—PCP 8

Given that the opioid crisis is of general medical concern, all PCPs were asked specifically about their thoughts on opioids prescribing for CNCP. Most declared to be very reluctant in prescribing opioids for their patients who suffer from CNCP, and some would only do so as a last resort.

“*So, if the previous therapy doesn’t work anymore and does not lead to success, then it is usually the case that the opioid is given as the next step. And then, as I said, you also check it again and see (…) if it works. That you start directly with opioids, that is rather rare, it’s rather if it’s actually patients whom you already know. So, if someone new comes to the practice with back pain, no matter how severe it is, I don’t really give an opioid straight away.*”—PCP 8

Some mentioned the fear that patients get dependent on opioids, and the difficulty to recognize such a dependency.

“W*e are talking about chronic pain, and it is of course difficult, there is also a certain tolerance, so that you then have to increase the dose again, sometimes you have to make a rotation of the opioids. That is quite difficult. And to what extent is this a dependency (…).—*PCP 6

Still, for a few PCPs there were patients and situations where an opioid treatment was useful and acceptable.

“*But I do have two or three patients with chronic pain, some of whom have been taking opioids for their pain for several years, in which [cases] the dose is quite constant, so I would not see this as opioid dependence in the sense of drug dependence. It is simply the most appropriate pain medication for this patient.*”—PCP 2

#### Non-pharmacological treatments

All PCPs also mentioned prescribing non-pharmacological treatments in order to reduce pain or help cope with a certain degree of it, including complementary medicine therapies. Physiotherapy was the most commonly mentioned first-line therapy, sometimes due to the fact that it is covered by basic health insurance, whereas complementary medicine like acupuncture is not, as was apparent in the previous theme.

In addition, some PCPs acknowledged that improving mental health had a positive effect on chronic pain, even if it was not to alleviate the pain, but rather to be able to learn how to cope with it. This relates back to the importance, underlined by some PCPs in the first theme, to investigate the psychological components of pain.

“*And depending on the situation, such a person may also need psychological or counseling support simply in order to deal with the pain (…). And to not carry this backpack [chronic pain] (…) as a huge backpack, but to carry it with them as luggage, but not to experience it as overwhelming, so that they actually learn coping strategies.*”—PCP 7

In addition to conventional psychotherapy, some PCPs recommended relaxation techniques, hypnosis, or meditation.

#### Reassessment

Independently of the treatment prescribed, to most PCPs it was important to reassess the situation regularly to check if there was a positive effect, and whether the treatment should be continued or discontinued. To assess the effectiveness of a treatment, some PCPs wanted patients to take it regularly over a certain amount of time.

“*I make sure that the patient, if they try it, take it consistently, in a dose that is reasonable, 2–3 times a day (…) for a period of 2–3 weeks. And that it can be determined afterwards, how did it help at all, does it help? Or did you realize, ok (…) that doesn’t help.*”—PCP 3

To most PCPs it was also crucial to assess the side-effects of certain treatments such as opioids.

“*I think you just have to check at the beginning whether it (…) helps and you have to question it critically whether it helps. Because if you obtain more side effects than [positive] effects, it doesn’t really help much.*”—PCP 8

#### Coping strategies

Finally, in addition to medication and therapies, PCPs highlighted the importance of coping strategies, or “learning to deal with the pain”. Most agreed that focusing on things other than pain might help patients to manage their daily life and to find meaning in it. One PCP explicitly told a patient to ignore the pain.

“*(…) And now I explained to the man, ’yes, well, now we have done what we can (…), you no longer have to be afraid (…) now you just have to live again, despite the pain’. And after some time, he actually went back to work. But it was just a long way. And he always wanted to come back and say ’The pain’ and I told him ’Yes, of course, the pain is there, but you just have to ignore it now.*”—PCP 1

## Discussion

Managing patients with CNCP in primary care is a complex task that requires PCPs to take many aspects into account, not only purely medical but also relational and psycho-social. The themes presented in the results were closely interconnected. Investigation, patient-provider relationship, and patient characteristics all potentially influenced the choice of a treatment, while patient-related factors may affect the investigation, the treatment, and the relationship. This complexity entails that PCPs need to figure out a unique strategy for each patient.

The importance of investigating CNCP and establishing a diagnosis has been pointed out in the past [32]. A recent scoping review [18] found that PCPs struggled with assessing and quantifying pain, as well as proposing a differential diagnosis. In the present study PCPs talked about unspecific or diffuse origin of chronic pain, making it difficult to treat. Other authors have highlighted a multifactorial etiology of CNCP, including pathophysiological mechanisms, patient history and social surrounding, as well as emotional and cognitive factors [8, 14, 19], which contribute to the complexity of managing patients suffering from chronic pain. All of these aspects were also mentioned by PCPs in this study. The difficulty to deal with patients’ request for specific investigations adds to this complexity and is exacerbated by barriers such as lack of time and social pressure [19, 21], which is consistent with findings of this study. Furthermore, PCPs generally considered the relationship to their patients as important and thus the need to preserve this relationship may also have an impact on the decision to undertake further investigations [16, 20, 21].

Another aspect that our study highlighted, that can be linked to the preservation of the patient-PCP relationship and was present in all four themes, is the importance of communication in pain management. Open, non-judgmental communication can impact patients’ adherence to their treatment as well as improve and maintain a collaborative relationship between patients’ and their PCP [33, 34]. While our participants did not explicitly link good communication with patients’ adherence to their treatments, they did insist on its importance in maintaining a good relationship, in explaining where chronic pain comes from and why it is not easily cured, and in understanding patients’ expectations and beliefs about pain, which may in turn have an impact on patients’ adherence. In addition, Hani and Liew [35] emphasized the difficulty of balancing physicians’ expectations of CNCP management with patients’ expectations, which demonstrates the importance of communicating about this issue.

For successful CNCP management, PCPs in this study took patients’ socioeconomic and psychosocial situation into consideration, thus adopting a biopsychosocial approach to chronic pain management that also takes social determinants of pain into account [36, 37]. The attention paid to these determinants was present at every stage of the treatment process and is recommended for better management of chronic pain [37]. For example, prevalence studies have shown the association of chronic pain with lower socioeconomic status, with an unclear relation of cause and consequence [1, 4, 6, 7]. Additionally, treatment costs and reimbursement by health insurance imposed a constraint on the choice of treatment, as others underlined [17], especially for complementary medicine treatments, as PCPs sometimes chose not to use treatments they considered useful because the patient’s insurance would not reimburse it. This type of constraints may lead to less effective pain management.

Perceptions about their patients’ attitude towards led a minority of PCPs to express doubts about some patients’ willingness to get better. This may have impacted the scope of action of PCPs and probably their own commitment to the patients [16]. A few went as far as to suspect secondary gain from chronic pain. PCPs of this study are not alone with this worry: several other studies that investigated the complexity of chronic pain management observed concern about secondary gain [38–40]. However, the concept of secondary gain is a controversial area, notably implying the false concept of a conscious process on the part of patients, and should be used with prudence [41]. It is certainly more important for clinicians to explore and work with the patient on associated factors of chronic pain [36, 37]. In addition, studies about patient engagement and self-management have found better treatment adherence and outcome if healthcare providers were actively supporting patients in the process, rather than passively sharing advice [34, 42, 43]. These considerations bring us back to the importance of the quality of patient-physician relationship, which is also key to successful patient-engagement [34, 43].

Concerning treatment requests, PCPs of this study always assessed the treatments’ risks and benefits but were generally open to patients’ preferences. The impact of patients’ requests for treatment has mostly been researched in the context of opioids [22, 44]. Requests for opioids were associated with negative impact on patient-provider relationship and consultation satisfaction [22, 44]. However, PCPs in our study did not specifically mention difficulties linked with patients requests for opioids.

Regarding medical recommendations, the interviews showed that most PCPs list a wide range of treatment options that they consider for their patients, ranging from pharmacological therapies, including opioids, to non-pharmacological ones, including complementary medicine. Each PCP had their own spectrum of therapies with which they were familiar and thus recommended and prescribed [19]. Altogether, our results show that PCPs seem to be very aware of the necessity of a biopsychosocial approach to CNCP, and were willing to consider patients’ needs and circumstances and adapt treatments accordingly, in accordance with recommendations on the subject [14, 45]. On the topic of opioid prescription, participating PCPs were generally rather critical about opioids and reserved them as treatment of last resort.

Finally, once a treatment was prescribed, PCPs highlighted the importance of revaluation, thus acting according to clinical recommendations about chronic pain care [8].

A particular strength of this study is to shed light on the whole process of managing patients with CNCP, without focusing on a given treatment option or pain site. Having the PCPs describe this process, from history taking to medical recommendations, allowed to underline its complexity and showed the interplay of the various factors PCPs have to consider in order to provide the best care for their patients. This study also has several limitations. First, the sample of PCPs that participated was rather small and from a specific part of Switzerland. It is therefore possible, that there are more aspects to the management of CNCP, that weren’t detected. Second, thematic saturation could not be achieved due to our small sample. As this study was carried out as part of a student’s (LR) Master’s thesis in medicine, the choice of the number of interviews to be conducted was constrained by the time available to LR to carry out the whole study. It was thus not possible to carry out more interviews to reach thematic saturation. However, when coding the last interviews, only few new codes were added to the codebook. These new codes did not affect the definition of the main subthemes and themes, which may indicate that thematic saturation had almost been reached. Finally, we have not explored any patients’ views in this study. Further research is needed to find out whether patients’ actual needs and resources are congruent with PCP’s assumptions.

## Conclusion

The results of this study highlight the complexity of chronic pain management. Psychological, social and economic aspects and their impacts on chronic non-cancer pain play a crucial role. PCPs are in a position to take the lead in CNCP management, through integrating patients’ values and beliefs, socioeconomic aspects of patients and health care insurances. Good communication about chronic pain and treatment options helps to align patients and PCPs expectations. It is the PCPs role to establish a trustful and non-judgmental relationship to improve patient participation. Considering the burden of this disease, more continuous medical education on chronic pain is needed for PCPs, especially to better take into account the various determinants of pain.

## Supporting information

S1 Appendix Interview guide. (TIF)
