# Supplementary figures and images for: Management of chronic non-cancer pain by primary care physicians: A qualitative study
Source: PLoS One. 2024 Jul 26;19(7):e0307701. doi: 10.1371/journal.pone.0307701 (PMC11280216; doi:10.1371/journal.pone.0307701)

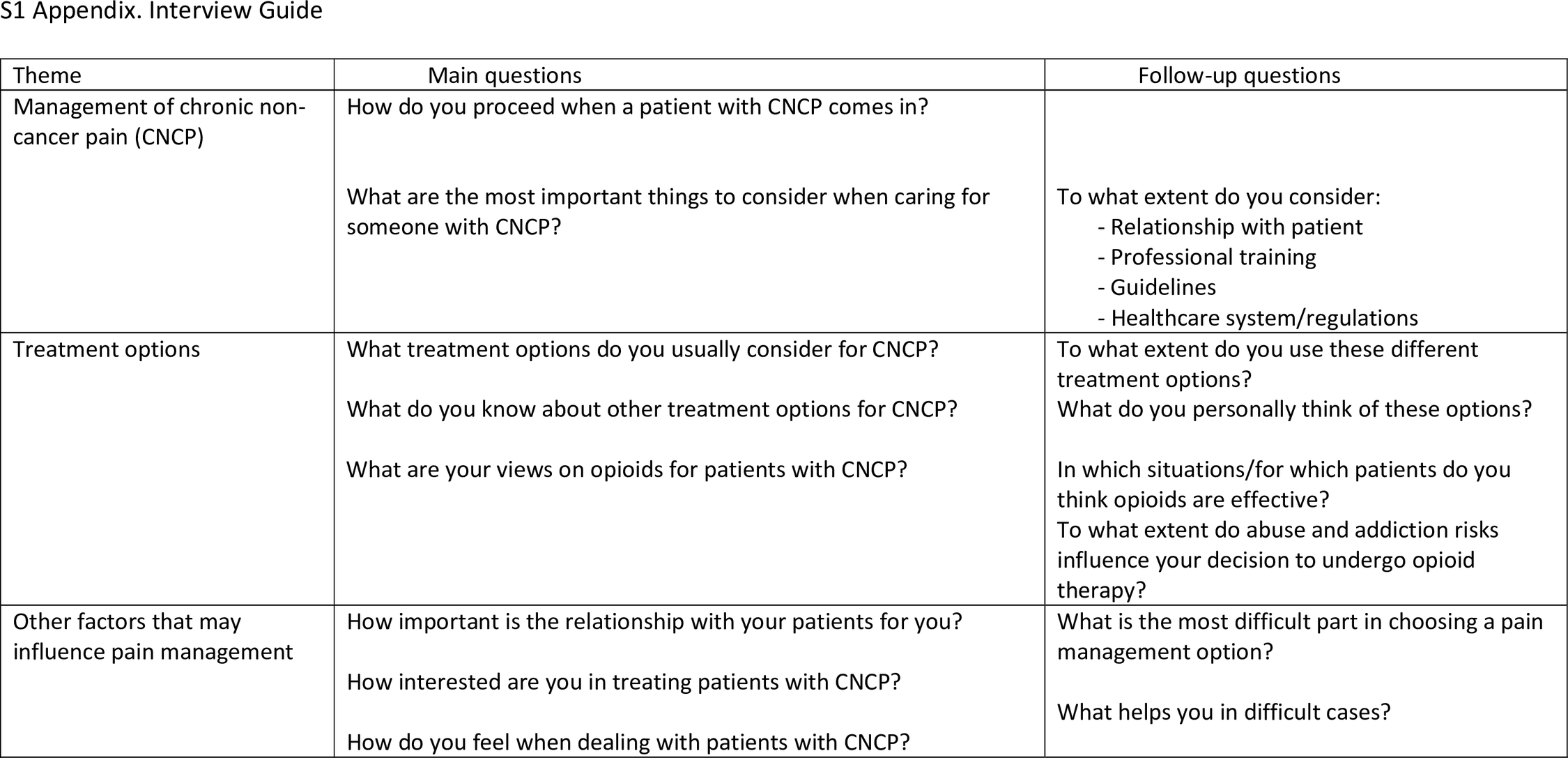

Supplement: S1 Appendix — (TIF) [file pone.0307701.s001.tif]
